# A global and regional view of the opportunity for climate-smart mariculture

**DOI:** 10.1098/rstb.2021.0128

**Published:** 2022-07-04

**Authors:** Heidi K. Alleway, Alice R. Jones, Seth J. Theuerkauf, Robert C. Jones

**Affiliations:** ^1^ University of Adelaide, Adelaide, South Australia 5005, Australia; ^2^ Provide Food and Water Sustainably Team, The Nature Conservancy, Arlington, VA 22203, USA; ^3^ School of Biological Sciences and Environment Institute, University of Adelaide, Adelaide, South Australia 5005, Australia; ^4^ Government of South Australia Department for Environment and Water, Adelaide, South Australia 5000, Australia; ^5^ Office of Aquaculture, National Oceanic and Atmospheric Administration National Marine Fisheries Service, Silver Spring, MD 20910, USA

**Keywords:** aquaculture, mariculture, climate change, climate change mitigation, food systems, resilience

## Abstract

Food systems and the communities they support are increasingly challenged by climate change and the need to arrest escalating threats through mitigation and adaptation. To ensure climate change mitigation strategies can be implemented effectively and to support substantial gains in greenhouse gas emissions reduction, it is, therefore, valuable to understand where climate-smart strategies might be used for best effect. We assessed mariculture in 171 coastal countries for vulnerabilities to climate change (12 indicators) and opportunities to deliver climate mitigation outcomes (nine indicators). We identified Northern America and Europe as having comparatively lower regional vulnerability and higher opportunity for impact on climate mitigation. Australia, Canada, France, Italy, Japan, Republic of Korea, New Zealand, Norway and the United States of America were identified as well-positioned to advance strategies linked to mariculture. However, the nature of vulnerabilities and opportunities within and between all regions and countries varied, due to the formation of existing mariculture, human development factors and governance capacity. Our analysis demonstrates that global discussion will be valuable to motivating climate-smart approaches associated with mariculture, but to ensure these solutions contribute to a resilient future, for industry, ecosystems and communities, local adaptation will be needed to address constraints and to leverage local prospects.

This article is part of the theme issue ‘Nurturing resilient marine ecosystems’.

## 1. Introduction

Food systems are highly exposed to the effects of climate change but are, in themselves, key contributors with 26% of total global greenhouse gas (GHG) emissions coming from industry associated with food production [1,2]. Strategies that can reduce the climate impacts of food production while meeting increasing demand are, consequently, critically needed [3]. Mariculture (aquaculture in marine environments) is an important and growing food sector and can form a lower GHG emission source of protein than agricultural sources (e.g. beef and pork) [2,4]. Emissions from finfish and shellfish aquaculture in 2017 (93% of global aquaculture production and excluding aquatic plants) were estimated to be 0.49% of total anthropogenic sources (263 Mt CO2e [5]). But this figure doesn't account for emissions from post-harvest activities and the supply of seafood to market, nor indirect emissions, such as those that might occur through degradation of the environment. Ongoing emissions associated with the mariculture production system are therefore undoubtedly higher, and while sustainable growth in this industry could make a valuable contribution to food and nutritional security [6–8], mitigation of emissions must be a focus in its development.

Mariculture is also garnering attention for its potential to support nature-based solutions, such as the farming of seaweed for GHG emissions reductions through its use in animal feeds—which can reduce methane—as biofuels, or as products that might support carbon sequestration, such as fertilizers that improve soil health and carbon retention [9,10]. Yet, mariculture—like all aquaculture—is highly susceptible to a range of climate impacts, including physical effects such as shifts in weather patterns and severe weather events [11–13], but also socio-economic capacity and the ability of industry, government and communities to respond to change [14–16]. It is anticipated that climate change will have some positive effects on mariculture, including enabling the growth of species in new locations or extended growing seasons, but the impacts on this industry are projected to be predominantly negative and to occur throughout the value chain [14,17,18]. Impacts are also expected to occur throughout culture environments (freshwater, brackish and marine), though the effects of some key threats, particularly severe weather events and changes to water quality (e.g. salinity, ocean acidification), will most certainly have a greater impact on coastal areas and aquaculture in brackish and marine environments [14,17]. Furthermore, aquatic foods are some of the most highly traded commodities among food and agricultural sectors. The exposure of supply chains to climate-related disruptions can exacerbate the vulnerability of mariculture to climate change [19,20].

To ensure that climate change mitigation can be achieved it is useful to understand how and where mitigation strategies can be deployed for maximum benefit. The vulnerability of food production systems and communities can, however, present a barrier to their implementation and sustainability, because they can, for example, demand additional resources not readily available or require industry to move or expand [16,17,21,22]. Here, we combine a view of the vulnerability of mariculture to climate change with an assessment of the opportunity to leverage a range of factors as pathways to climate change mitigation. Twelve indicators of vulnerability from five themes and nine indicators of leverage opportunity from four themes were used to assess 171 coastal countries, and the implications of the assessment explored at global, regional and country scales (box 1). This study builds on existing analyses, such as integrated assessments of fisheries and aquaculture and their relationship with sustainability objectives (e.g. [27]), by considering a broad range of factors explicitly associated with the mariculture production system and ways in which this industry could be linked to emissions reduction and climate mitigation strategies. To rise to the challenge of meeting the growing demand for food within increasingly constrained environmental limits [8], development of a climate-smart mariculture industry at successive spatial scales must occur [17]. Our analysis assists in understanding which countries are currently well-placed to implement climate-smart mariculture strategies, and the strategies that may be available to all coastal countries worldwide to realize climate mitigation outcomes, in light of the vulnerabilities they will face as a result of climate change.

Box 1. **Interpreting vulnerabilities and leverage for climate-smart outcomes in mariculture.**

We collated indicator data on the vulnerability of mariculture to climate change impacts and the opportunities associated with development of a climate-smart mariculture industry. The intention is for these data and analyses to support industry sectors, governments and international organizations to respond to climate change impacts on, and from, mariculture, and to pursue climate-smart strategies. These responses may be specific to a country or region, or they may bridge challenges across multiple jurisdictions.

***Example 1: Cross-jurisdictional design, seafood consumption***

Countries with high rates of consumption and importation of fish and fishery products (greater than the 80th percentile of the dataset based on all countries, e.g. France, Fiji, Kiribati) could implement strategies to decrease the proportion of imported products in favour of increased domestic production, thus reducing the length of supply chains and associated GHG emissions and building resilience in domestic mariculture. Indicators of the scale of opportunity to implement this strategy include an understanding of the vulnerability of consumption (reliance or preference for fish and fishery products) and a measure of the countries' seafood consumption footprint. Promoting local products in domestic markets (e.g. building brand value for provenance, food safety standards or values, tax incentives) could be used to support growth in use and subsequent production of local food. However, where a country is also ‘vulnerable’ due to, for example, *low* rates of annual production (under the 20th percentile) or diversity in the production portfolio, operational strategies such as increased investment into research, infrastructure, supporting legislation or training and development will be needed to support this objective. This example is illustrated in countries such as Timor-Leste, where the opportunity to reduce reliance on imported products is *high* (the country is currently above the 80th percentile for this metric) and vulnerability in production is *medium* (between the 40th and 60th percentiles). However, vulnerability is increased by a lack of evenness (less than 20th percentile) in the number of different species produced, which could see regular shocks to the production system reducing the country's capacity to reliably meet greater domestic demand.

***Example 2: Country-scale analysis, Japan***

When evaluating vulnerabilities (impacts of climate change on mariculture at the country-scale reported in electronic supplementary information, figure S1), Japan exhibits *high* levels of seafood consumption *per capita* (i.e. greater than 80th percentile based on all countries' data*) with *medium* variance (between the 40th and 60th percentiles based on data from all countries), rendering the population and export commodities vulnerable to fluctuations in seafood trade and supply. However, this risk is offset by low vulnerability to the impacts of climate change in domestic mariculture production, characterized by high production quantities (greater than 80th percentile), *low* variance in production (less than 20th percentile of data from all countries) and high diversity across mariculture sectors and the species produced (the production diversity value is greater than 80th percentile of the full dataset), which can indicate greater stability and resilience [20,23]. Japan also has *low* and *low–medium* vulnerability scores for projected changes to finfish production from climate change and for the GFSI Natural Resources and Resilience score (less than 20th percentile and between the 20th and 40th percentiles respectively for the relevant datasets). That said, the coastal waters of Japan are at a *high* risk from coastal eutrophication (scoring above the 80th percentile for coastal nutrient pollution), an environmental stressor that can exacerbate climate change effects, such as warming waters, and reduce the marine environment's overall resilience [24].

Leverage opportunities for Japan appear to be diverse and numerous (opportunity for levereage at the country-scale reported in electronic supplementary information, figure S2), suggesting a suite of strategies may be available to realize the development of climate-smart approaches. Reducing imported seafood by increasing domestic aquaculture production may present an opportunity to decrease the footprint of seafood consumption by shortening supply chains, thereby reducing GHG emissions associated with post-farming activities (e.g. offshore processing and transportation). Although high quantities of emissions can typically be attributable to production (e.g. emissions associated with on-farm activities) [5], increasing globalization of seafood products and trade (e.g. importation of feed, re-importation of raw or value-added product previously exported or export of products to remote markets) [23] might undermine otherwise low emissions profiles. Strategic development of bivalve shellfish and seaweed aquaculture to promote ecosystem recovery (e.g. nutrient removal [25,26]) could provide a valuable leverage opportunity for Japanese mariculture, contributing to reducing coastal eutrophication and in turn increasing local marine environmental resilience to climate change impacts and cumulative stressors. Japan scores *high* for leverage opportunity through factors that can enable mariculture development and adaptation (greater than 80th percentile based on all countries' data, for each indicator), including sound regulatory quality, logistics performance and investment into research and development, indicating the country is well-placed to implement practices that proactively address climate risk and grow positive climate outcomes from industry activity.

*Percentiles used to classify indicator data into measures of vulnerability and leverage opportunity (low through high), provided in parentheses, are included as *examples and apply to the country and regional descriptions of indicators classifications throughout §3. See Materials and Methods, and Supplementary Methods and Supplementary table 1 in the electronic supplementary material for description of the designation of ‘low’, ‘low–medium, ‘medium’, ‘medium-high’ and ‘high’ classifications*.

‘Vulnerability’ in our analysis refers to the collective impact of climate change on mariculture, and the resulting limitations that countries may face in sustaining or growing this industry into the future. Our use of this term incorporates, but does not isolate, measurement of exposure, sensitivity and adaptive capacity, which are commonly used in climate vulnerability assessments [14,15,28]. The indicators used relate to mariculture as a food production system (e.g. species and quantities produced, consumption of seafood products, governance) and are consistent with the types of responses needed to build industry and community capacity to respond to climate change, specifically absorptive capacity (capacity for persistence), adaptive capacity (incremental adjustment) and transformative capacity (transformational responses) [29,30]. Our view of these types of capacity follow the definition of these factors provided by Béné *et al*. [30], and resilience is the result of maintaining and improving each of these types of capacity in an integrative way [30]. Prior assessments of the impacts of climate change on aquaculture highlight that adaptive capacity, in particular, will be influential in the successful adaptation of climate mitigation strategies, and should be implemented in parallel with strategies that support broader adaptation outcomes [14,17].

‘Leverage’ describes pathways through which climate mitigation approaches could be focused, because ‘leverage points’ can be used to identify places where transformation for sustainability can be realized in complex food systems [31]. For example, interventions for reducing climate impacts from industry practices can include enhancing efficiencies in crop yields or converting crop production for human food use (rather than animal feed), thereby reducing calorific loss from food waste [32]. To date, mariculture has been largely excluded from the narrative of sustainable global food production (i.e. viewing aquaculture as a food system) despite many aspects of mariculture being synonymous with agricultural practices, such as the use of feed and production of waste [33,34]. The importance of seafood in food and nutritional security means its inclusion in approaches to broader food policies to increase resilience and our responses to climate change is needed [35].

## 2. Methods

### (a) Data collection and processing

Inconsistent reporting in datasets occurs across seafood industries, and there is known to be sustained misrepresentation of mariculture statistics at various scales [14,33,36]. Aquaculture data in global datasets can also be over-aggregated in comparison to fisheries data (for example, the Organisation for Economic Co-operation and Development provides country-level aquaculture production data but only in aggregate, by combining freshwater and marine culture as well as multiple organisms, despite more explicitly detailing fisheries statistics), thus precluding the use of valuable sources of information in climate mitigation analyses. Additionally, databases that do contain detailed information relevant to mariculture are not always identifiable via conventional search methods and search terms. An important example is the UN Food and Agriculture Organization's FishStatJ database [37], which provides access to the most recent and complete seafood production and consumption statistics. Despite being widely known and regarded, this database does not appear as an obvious dataset under many search terms and can be overlooked in a structured review. To approach this complexity of disparate data sources and varying comprehensiveness, we used an iterative search process to identify indicators of vulnerability and leverage and associated datasets. This process was informed by an understanding of the types of data and databases used in the literature to inform similar climate and aquaculture analyses and the expert opinion of the authors through a series of workshops directed by structured questions and resolutions. During these workshops, information and datasets for potential indicators were reviewed, decisions on indicators to be included and excluded at each step in the exploratory process were resolved, and the direction of the relationship of the indicators was agreed.

To determine the indicators used, we first identified high-level themes considered most relevant to climate change vulnerability (food production, seafood consumption, climate change impacts, development status and resource resilience) and the opportunity to leverage gains in GHG emissions (potential for GHG emissions reduction, ecosystem services, production and supply chains and leverage enablers associated with governance and adaptive capacity). These themes represent key factors associated with different parts of the mariculture production system. But the impacts of climate change, and pathways to mitigate its effects, are also influenced by social and economic factors, hence a range of indicators associated with interlinked social and economic vulnerabilities and mitigation approaches were therefore also included (e.g. national seafood consumption, governance and adaptive capacity). Drawing on examples from the literature, a broad list of indicators that could potentially be used to assess more discrete aspects of these themes was then generated. Each potential indicator was screened for available data that could enable assessment to a country-scale. To foster access and visibility of the existing information on mariculture and climate change, we favoured the use of open source, readily available datasets, in particular United Nations and World Bank data, including Food and Agriculture Organization (FAO) statistics for aquaculture production and consumption of fish and fishery products, and the Human Development Index (HDI) and Global Food Security Index (GFSI). Composite scores or indices were also favoured for some indicators, such as projected climate impacts, because these indices would facilitate the consideration of a range factors within that dataset (e.g. the GFSI includes data on a range of factors associated with food affordability, availability, quality and safety, and Natural Resources and Resilience; the projected climate impacts in Froehlich *et al*. [38] include consideration of sea surface temperature, chlorophyll and ocean acidification [38]). From this process, 29 indicators of vulnerability (*N* indicators = 15) and leverage (*N* indicators = 14) were identified as highly relevant and available for analysis. Data on all 29 indicators were collated and a pairwise test was used to check for correlation. Eight indicators were excluded on the basis of being highly correlated with other similar indicators (greater than 0.8), or due to there being insufficient resolution in the dataset to enable assessment to a country-scale (see electronic supplementary information for methods and results associated with the correlation test and description of the indicators excluded). In addition, where the length of the time-series and comprehensiveness of the data permitted, we initially generated summary values for each indicator over two distinct time periods (2000–2009 and 2010–2017). Data for the two time periods were found to be strongly positively correlated for all indicators assessed. The results presented in the final analysis were, therefore, based on the later time period only and these values should be viewed as also representative of the data for the first time period. Twenty-one indicators were adopted for final analysis (table 1).

**Table 1.** Indicators of (1) vulnerability of mariculture from climate change, and (2) opportunities for leverage in the development of climate-smart approaches in mariculture. Table includes data sources and raw data units, rationale for inclusion, data processing, steps and the direction of the relationship between the indicator and vulnerability or leverage (see supplementary information for additional description and methods used for the indicators adopted).

**#**	name	rationale for inclusion in assessment	reference and source of data	data and processing methods	raw data units	direction of relationship with vulnerability
(1) vulnerability indicators						
*food production*						
1.1.1	mean annual production	strong (higher) production quantities reflect capacity to engage in aquaculture and the basis from which production may be increased or modified (i.e. builds resilience to climate change impacts). Variability in production can increase the negative impact of shocks as it is indicative of less consistent supply or demand and/or a less stable aquaculture industry in general.	FishStatJ, FAO global fishery and aquaculture production statistics [37]	average of total annual production all sectors, 2010–2017	tonnes live weight	−
1.1.2	variance in annual production			coefficient of variation of mean annual production, 2010–2017	tonnes live weight	+
1.1.3	mean annual production *per capita*			average of total annual production across all sectors divided by annual population, 2010–2017	tonnes live weight *per capita*	−
1.1.4	diversity of species produced	a diverse production portfolio can make overall production tonnage more stable over time, reducing volatility in supply and markets, and contributing to resilience in the face of the impacts of climate change		Shannon's *H* diversity calculated from species-specific production tonnages, 2010–2017	Shannon's *H* index	−
1.1.5	evenness of species produced			Pielou's *J*, based on species diversity and production tonnage data, 2010–2017	Pielou's *J* index	−
*seafood consumption*						
1.2.1	mean annual consumption of fish and fishery products *per capita*	high rates of consumption can indicate reliance on fish and fishery products for food and markets, and greater potential exposure to shocks from climate change impacts	FAOSTAT, FAO Food Balances seafood consumption statistics [39]	mean annual apparent consumption of marine fish and fishery products *per capita*, 2000–2013	kg *per capita* per year	+
1.2.2	variance in annual consumption *per capita* of fish and fishery products	variability in consumption may indicate underlying vulnerability associated with access to food (e.g. food security, economic security, equality)		coefficient of variation of mean annual apparent consumption of fish and fishery products *per capita*, 2000–2013	kg *per capita* per year	+
*climate change impacts*						
1.3.1	projected climate change impact – bivalves	aquacultured species growth patterns and overall marine productivity will, in many cases, be negatively impacted by the biophysical effects of climate change, leading to vulnerability in aquaculture production	data from analysis in Froehlich, Gentry and Halpern [38] of global projections of change in aquaculture production under climate change (see electronic supplementary data) [38]	published data on projected probability of changes in production of bivalves to 2050 applied	percent decline inproduction	+
1.3.2	projected climate change impact – finfish			published data on projected probability of changes in production of finfish to 2050 applied	percent decline in production capacity	+
*development capacity*						
1.4.1	human development index score	human capacity, particularly low income and inequality, can increase the exposure of communities and effects of shocks associated with climate change and therefore increase vulnerability to climate change impacts	Human Development Index, UN composite score [40]	index score for 2017	index	−
*resource resilience*						
1.5.1	impact from coastal eutrophication	coastal eutrophication is a primary driver of poor coastal water quality and contributes to cumulative impacts in marine environments, which may limit adaptation or recovery/resilience	data from Hoekstra *et al*. [41], change in discharge of nitrogen to the coast during the pre-industrial to contemporary time period [41]	trend in change in discharge of inorganic nitrogen to the coast normalized to the area of a country's Exclusive Economic Zone	Tg per year	+
1.5.2	global food security index, Natural Resources and Resilience score	climatic patterns and events have persistent chronic and acute impacts on production that will vary in frequency or intensity as a result of climate change; the higher the resilience of a country's natural resources is, the lower the vulnerability to these impacts	Global Food Security Index, The Economist Group annual composite score [42]	index score for 2018	index	−
(2) leverage indicators						
*GHG emissions reduction*						
2.1.1	opportunity to reduce CO_2_ emissions	*per capita* emissions indicate the degree of opportunity for each country to target reductions in GHG from climate-smart aquaculture	CO_2_ and GHG emissions, World Bank data [43]	average of annual CO_2_ emissions *per capita*, 2010–2017	tonnes per year *per capita*	+
2.1.2	opportunity to reduce seafood consumption footprint	high proportions of imported product, long supply chains and complex production cycles for seafood consumption indicate the degree of opportunity for each country to target reductions (e.g. in supply chain stages, market locations), which are directly related to reducing emissions and the vulnerability of supply to climate change impacts	data from Guillen *et al*. [44], global seafood consumption footprint (electronic supplementary material)	published data measuring ‘seafood consumption footprint’ – biomass of domestic and imported seafood required to satisfy a country's consumption (aquaculture product plus fish meal, but excluding wild-caught fish for consumption) in 2011 – applied	kg *per capita*	+
2.1.3	opportunity to reduce proportion of imported seafood product	in addition to consumption, the proportion of imported seafood product may indicate potential to decrease imports and increase local market demand and domestic supply from comparably lower GHG emissions sources (sectors, businesses) (with potential for associated benefits of reduced GHG emissions and boosting local economies and community resilience)	FishStatJ, FAO global fishery and aquaculture production and fish trade statistics [37]	slope coefficient from a linear model of a country's ratio of total aquaculture production to imported seafood over time, 2000–2017	ratio (total aquaculture production to quantity of imported seafood, tonnes per year)	−
*ecosystem services*						
2.2.1	potential for restorative aquaculture – seaweed	delivering co-benefits from targeted actions can increase the effectiveness of climate-smart practices and support multiple objectives for resilience (e.g. mariculture that can simultaneously support food and ecosystem outcomes and help reduce local climate impacts)	data from Theuerkauf *et al*. [25], global analysis of Restorative Aquaculture Opportunity Index (electronic supplementary information) [25]	index score indicative of opportunity to benefit from ecosystem services provided by restorative seaweed aquaculture applied	index	+
2.2.2	potential for restorative aquaculture – bivalve shellfish			index score indicative of opportunity to benefit from ecosystem services provided by restorative shellfish aquaculture applied	index	+
*production and supply chains*						
2.3.1	opportunity to increase production from low GHG emissions sectors	facilitating increases in low GHG emissions sectors may support GHG emissions reduction and mitigation policies	FishStatJ, FAO global fishery and aquaculture production statistics [37]	average annual proportion of aquaculture production coming from marine mollusc and aquatic plant sectors (combined) in relation to total marine production, 2010–2017	percentage of production	−
*leverage enablers — governance and adaptive capacity*						
2.4.1	regulatory quality	good governance increases capacity for adaptation as well as development opportunities and implementation of sustainability goals	World Governance Indicator, Regulatory Quality score [45]	index score for 2018	index	+
2.4.2	logistics performance	good logistics enable access to seafood and supplying markets (domestic and export) and increase capacity to adapt to production shocks and impacts of climate change	Logistics Performance Index, World Bank World Development Indicator score [43]	index score for 2018	index	+
2.4.3	research and development investment	investment into research and development supports development opportunities and capacity to adapt to climate change by using innovative approaches to build resilience into aquaculture food supply systems	research and development expenditure, World Bank data [43]	mean research and development expenditure as a percentage of GDP, 2010–17	percentage of GDP	+

### (b) Indicators of vulnerability

To assess mariculture in each country, data were collated on the portfolio of each country's production between 2000 and 2017 (marine fishes, diadromous fishes, crustaceans, molluscs, aquatic plants and miscellaneous animal species) from the FAO Global Fishery and Aquaculture Production Statistics v.2019.1.0 (using FishStatJ, v.3.05.3). From these data we derived measures for the mariculture production indicators, specifically: mean annual production (indicator 1.1.1), variance in production (coefficient of variation of the mean annual production in a given time period; 1.1.2) and mean total aquaculture production *per capita*, as a basic illustration of the scale of production within a country relative to its population (1.1.3). A more diverse food production portfolio can make production more stable over time, which can increase resilience [19,20]. Aquaculture is a diverse activity with a large number of species produced at a global level. However, the type and number of species produced across all countries vary considerably. We measured a country's diversity in production across different mariculture sectors (1.1.4) using the Shannon's *H* diversity metric [46] (see electronic supplementary information for further description of methods used to assess each indicator), based on species richness and annual total production quantities for each species from the FAO production data. As well as assessing the diversity of the production portfolio, we also considered a country's ‘evenness’ in production, to provide insight into countries where the portfolio may be diverse in terms of the total number of species produced but still vulnerable to shocks, because production is disproportionately biased toward high volumes from a small number of species. Pielou's *J* evenness metric [47] was used to assess this indicator (1.1.5), using the maximum number of species produced in a region as the denominator. Adopting the maximum number of species within a region for this measure, rather than the global maximum, prevented a disproportionate influence from countries from other regions that produce very small or very large numbers of species, and therefore the likelihood that regional trends in evenness would be obscured (e.g. by comparing the diversity of production in Asian countries with that of small island nations in the Southwest Pacific).

Seafood is a popular commodity and an important source of protein and nutrition. This can expose countries with a dependence on seafood to production- or trade-related shocks [19]. The FAO Food Balance Sheet dataset [39] was used to generate mean apparent consumption of fish and fishery products (kg) *per capita* per year (1.2.1) and variability around the mean (1.2.2), for the two time periods 2000–2009 and 2010–2013 (2013 being the last year of data available at the time these data were downloaded).

Climate change impacts to aquaculture production were considered based on data from a recent study [38], which projected changes in capacity of coastal aquaculture sectors under altered temperature and pH conditions. We used country-level projections to 2050 of the percentage change in production, positive or negative, from this study for bivalves (1.3.1) and finfish (1.3.2).

The HDI score for each country (1.4.1) was included as an indicator of human capacity, and therefore vulnerability [30], using the most recent assessments of HDI data available (2017). To understand patterns associated with declining or unchanged HDI scores for countries that had *high* and *medium-high* vulnerability, we also assessed their trend in HDI scores from 1990–2017. This was done by extracting the slope coefficient from a linear model of the HDI score over time, excluding countries with less than three years of data. Smaller coefficient values represented a stagnation of the HDI score, or a smaller change over time, thus providing an indication of countries that may be particularly at risk from both low HDI scores (those ranking as high or medium-high vulnerability) and unchanged or minimal progress in realizing human development outcomes (see also electronic supplementary information and Results). Because this assessment was only undertaken for countries scoring *high* and *medium-high* for HDI score, we did not include the HDI trend classification in the primary results, but rather as a supplemental analysis (see electronic supplementary information, table S5).

To understand threats to the resilience of coastal natural resources, we used a measure of long-term change in the discharge of dissolved inorganic nitrogen to coastal areas (1.5.1). Coastal eutrophication is a key contributor to cumulative effects that can decrease ecosystem health and reduce resilience, and will compound the impacts of climate change [24]. The overwhelming majority of mariculture occurs in the coastal zone, and so this threat is especially relevant to industry activity in this environment. Eutrophication data were normalized according to the area of a country's Exclusive Economic Zone to provide a more repetitive measure of this threat at a country-scale.

The composite GFSI score was also included as an indicator because this index measures a country's overall food security based on a variety of contributing sub-scores of exposure in the natural assets crucial to food security; water, land and oceans. The natural resource and resilience scores (Category 4 of the GFSI index) are a risk adjustment factor representing a country's exposure to climate impacts and included measures of exposure to 28 factors, such as changes in temperature, drought, flooding, storm severity and sea-level rise. We used the natural resource-related risk and vulnerability score as an indicator to incorporate an understanding of broader climate change influences in the ocean and on land (1.5.2), because land-based effects, such as drought, can generate impacts to aquaculture where there is a reliance on terrestrial inputs (e.g. impacts on crops used for feed or runoff through catchments) [27].

### (c) Indicators of leverage

Annual *per capita* CO_2_ emissions were obtained from the World Bank, ‘Our World in Data’ (2.1.1), and summarized to generate a mean for each country over two time periods, 2000–2009 and 2010–2017. Countries that have greater CO_2_ emissions *per capita* provide a prospect to target transformative change in GHG emissions reductions. As a highly traded commodity, the consumption and production of seafood also require consideration of associated GHG emissions and opportunities for reductions (e.g. reductions in ‘food miles’). We included scores for the seafood ‘consumption footprint’ (kg *per capita*) generated in a recent study [44] that account for multiple inputs and outputs, including the use of feed for aquaculture. An aggregated score of the consumption footprint that measures aquaculture and fish meal (as a requirement for the production of some aquaculture products), but excluding measurement of the footprint of wild-caught fisheries consumption, was adopted (2.1.2).

Improving supply chains that may be GHG emissions-intensive could provide a leverage point to close the ‘diet gap’ [32]. Increasing consumption of domestic mariculture products may therefore present an opportunity to reduce GHG emissions by, for example, reducing excessive transport of products (e.g. importation of locally produced feed) or the importation of goods that can be produced locally. As an indicator of a country's gross mariculture supply chain, and the opportunity to reduce supply chain emissions, we calculated the proportion of domestic mariculture production in relation to imported seafood products (using the FAO data) for each year from 2000–2017. We assessed changes in the proportion of production versus imports over time using the slope coefficient from a linear model of each country's proportional values. The slope coefficient was used to indicate the direction (+ or −) and the magnitude of any trend in production:import quantities through time (2.1.3).

The potential to leverage environmental co-benefits from mariculture through ecosystem services was considered using the global Restorative Aquaculture Opportunity Index [25] for seaweed (2.2.1) and bivalve shellfish production (2.2.2). This index accounts for a range of local environmental, socio-economic and human health factors that create impacts to marine areas and can enable restorative aquaculture outcomes. We also assessed the potential for growth in low emissions aquaculture sectors (a reduction associated with ‘yield gap’ [32]), using the average of the proportion of a country's mean total mariculture production per annum for the aquatic plant and mollusc aquaculture sectors (2.2.3). Life cycle analyses of sector or species-specific GHG emissions for aquaculture have identified aquatic plant and mollusc aquaculture, particularly bivalves, as having comparably low GHG emissions in comparison to other sectors [1,4,48].

The quality of governance—its scope and useability—can influence how vulnerable the aquaculture industry is to climate change [28]. Regions and countries that are more prepared for or more capable of responding and adapting to the impacts of climate change will have better capacity to implement ongoing strategies in aquaculture production systems, which will reinforce improvements in resilience through positive change in the absorptive, adaptive and transformative capacity of a community [30]. Good governance and investment into infrastructure, knowledge and capacity can support effective, sustainable development [49,50]. We viewed these factors as ‘enablers’ of this opportunity, and therefore leverage. The capacity to leverage the existing status of these key enablers was assessed using indicators of overall regulatory quality (2.4.1) and logistics performance (2.4.2) from the World Bank (the World Governance and Development Indicators), and research and development expenditure as a percentage of a country's gross domestic product (2.4.3).

### (d) Analysis and visualization

Data processing and visualization were undertaken using R statistical software [51]. The FAO's system of regional and country classification was used to aggregate data for assessment, focusing only on coastal countries (i.e. those with opportunity for mariculture). A semi-qualitative approach is commonly adopted in aquaculture vulnerability assessments due to limitations in the comparability of raw data across factors and numeric scales. In this approach, a range of datasets that can measure sensitivity, exposure and adaptive capacity are often pooled and classified into ranks or scores to generate more comparable metrics [14,28]. The datasets used in this study required the same approach, and it enabled us to consider vulnerability and leverage using a consistent measure. Our analysis compared data across the countries assessed rather than measuring the actual effect of a vulnerability and leverage indicator *per se* (e.g. a measure of the effect of climate change on production). This approach further enabled comparison at multiple spatial scales, but the results must therefore be viewed as relative to the cohort of countries assessed and the indicators used. Additionally, we sought to understand the opportunities available for directing climate-smart approaches, hence our interpretation of ‘vulnerability’ and ‘leverage’ may differ from the interpretation of these factors in a study with a different focus, such as human development. For instance, depending on the context, high mariculture production quantities can be interpreted as making a country more vulnerable to climate change, because the degree of exposure and sensitivity to climate change can increase with higher production volumes [14,16] or, as per our assessment, more resilient and adaptive, contributing to absorptive capacity and improving the ability to recover from impacts. Weighting of the indicators used was considered during development of the study with a number of methods explored, but a weighting was not applied due to the difficulty in using this approach in a consistent way that is relevant at multiple spatial scales (global- to country-level), and the risk of inappropriately weighted indicators having an undue influence on the assessment.

To enable comparison across indicators and datasets we rescaled all data using a linear approach, converting raw data to a common numerical scale. This changed the lowest value of each indicator to 0 and the highest value to 10, rescaling all other values proportionately, such that the distribution of the dataset and the relative differences between country scores were preserved. During re-scaling, indicators with an inverse relationship to vulnerability and leverage were reversed to align all datasets along a common scale, with 0 being the ‘worst’ score (highest vulnerability or lowest leverage opportunity) and 10 the ‘best’ (lowest vulnerability or highest leverage opportunity). Re-scaled values for each indicator and aggregate regional scores, based on median values from all countries in a given region, were then classified according to five groups (*low, low–medium, medium, medium–high* and *high*) using the quantiles of the data distribution and break points every 20th percentile (see electronic supplementary information, table S1). Classification using quantiles was preferred over a cluster-based approach, due to some indicators being affected by outlying high values in a small number of countries (e.g. China's extremely high mariculture production quantities in comparison to other countries).

The distribution of country and regional indicator scores of vulnerability and leverage were explored using scatterplots of median values and the median classified indicator scores (*low* through *high*) and country and regional heat maps. We also identified countries that were consistently classified as either *high* or *low* for vulnerability or leverage, based on a threshold of consistency. Each region was assigned an overall ranking for their relative aggregate score across all vulnerability and leverage indicators, with ‘consistency’ defined as ≥ 40% of indicators being classed as high or low for vulnerability or leverage (see details on optimizing this threshold in the supplementary methods). By mapping the consistency of scoring we were able to look for spatial patterns, and identify countries and regions that were, for example, particularly vulnerable or of a high leverage position.

## 3. Results and discussion

### (a) A global view of vulnerability and opportunity

**Figure 1.**
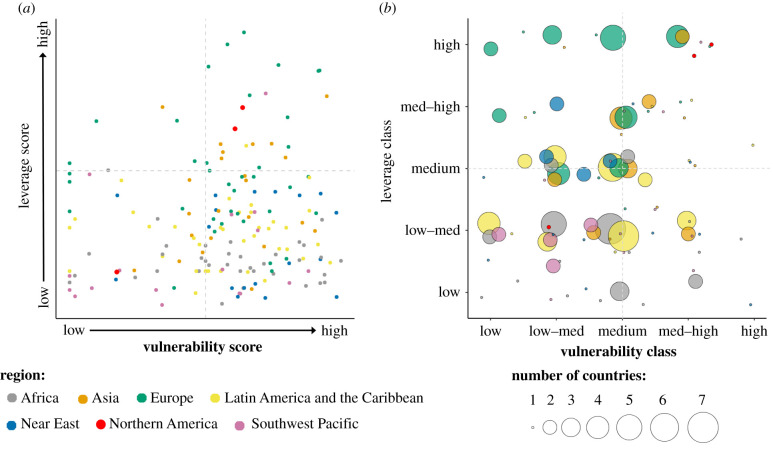
(*a*) Median vulnerability and leverage indicator scores for each country coloured by FAO region (vulnerability *N* = 12; leverage *N* = 9) and (*b*) combinations of median indicator classes, *low* through *high*, for leverage and vulnerability, displayed by the number of countries with each class combination (indicated by point size) within each FAO region (indicated by point colour).

Our results highlight that there may be opportunities to leverage climate-smart outcomes associated with mariculture throughout most of the world, both in countries that have had sustained high production over a long period of time and countries that have little-to-no existing mariculture activity. However, the number and nature of these opportunities differ markedly within and between regions, along with each country's vulnerability (figure 1*a*). Across all countries, median classified scores for all indicators tended to fall into the *low–medium* and *medium* classes (figure 1*b*). Europe, Northern America and to a lesser degree Asia, had a higher number of countries exhibiting median classifications of *medium*, *medium-high* and *high* across all indicators. This result emphasizes that while global discussion on climate-smart approaches could motivate and facilitate the design of industry-wide applications, effective responses will require locally contextualized solutions, to address needs, constraints and best leverage local prospects [52–54].

**Figure 2.**
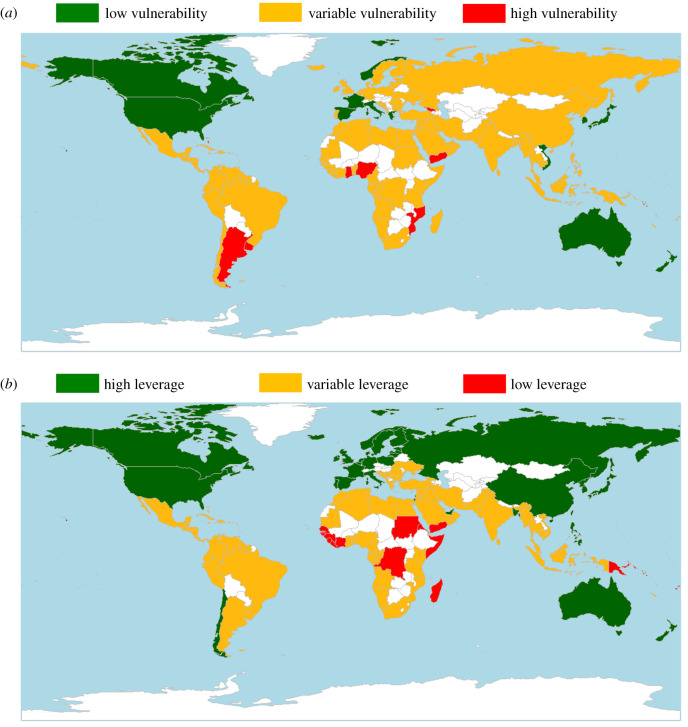
Countries scoring as consistently ‘high’, ‘low’ or ‘variable’ for (*a*) vulnerability of mariculture to climate change, and (*b*) opportunities for leverage in mariculture for climate mitigation. Categories of consistency are based on the proportion (greater than or equal to 40%) of indicators where a country was classified as ‘high’ or ‘low’. Countries not consistently classified as ‘high’ or ‘low’ across multiple indicators display as ‘variable’.

**Figure 3.**
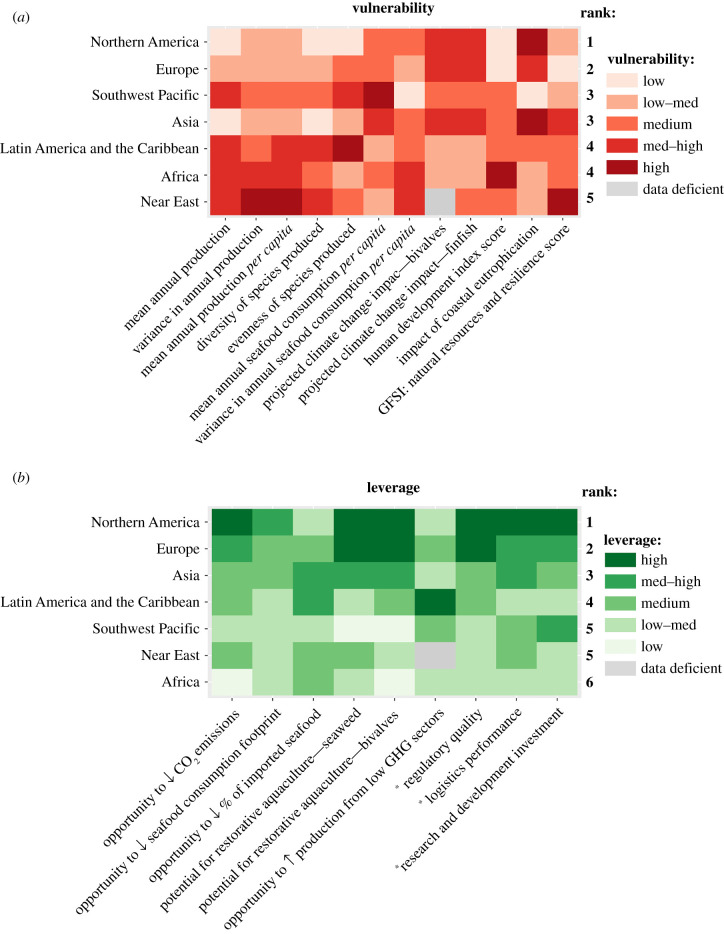
Regional summaries of indicator scores and relative ranking of all regions for (*a*) vulnerability of mariculture to climate change (least vulnerable to comparatively most vulnerable), and (*b*) opportunities to leverage mariculture to increase resilience and generate climate-smart operations (most opportunity to comparatively least opportunity). Regional indicator scores are based on the median value for each indicator, calculated using data from all countries in the region and classified (*low–high*) according to the quantiles of the full, country-resolution dataset. A region's ‘rank’ identifies the overall position of that region in comparison to others, taking account of all indicators. *Identify indicators that enable leverage opportunities.

A small cohort of countries with consistently *low* vulnerability and *high* opportunity for leverage may be better positioned to implement the climate change mitigation pathways assessed here, and potentially more likely to generate good outcomes from climate mitigation strategies in the short term (figure 2*a,b*, and electronic supplementary information, table S6). These countries—Australia, Canada, France, Italy, Japan, Republic of Korea, New Zealand, Norway and the United States of America—could benefit from an immediate focus on developing climate-smart mariculture, and generate higher, globally valuable outcomes. Many of these countries are among the world's highest GHG polluters; the USA, Australia and Canada ranking 1st, 2nd and 3rd respectively for CO_2_ emissions *per capita*, with Japan 5th and France 10th [55]. Amidst growing concerns over continued increases in the rate and magnitude of climate change, these countries must invest in widespread and immediate climate change mitigation solutions, including solutions linked to food systems. Using their *low* vulnerability and *high* leverage position (figure 3*a,b*) they could also provide regional and global value to the growth of climate-smart mariculture by leading the development, testing and advocacy of the underwriting mechanisms needed to support climate solutions throughout seafood industries, such as R&D investment, market development, advancements in feed and technology, and policy [6].

Countries identified as not having either consistently *high* or consistently *low* vulnerability or leverage represented a large cohort. In these ‘variable’ countries (figure 2*a,b*) certain aspects of the mariculture production system may be particularly vulnerable to climate change, or a smaller number of opportunities may be available for leverage (electronic supplementary information, figure S1 and figure S2). For instance, a country may have relatively high mean annual production (associated with *low* vulnerability in this indicator), adding a degree of resilience or adaptive capacity to the production system despite higher projected impacts from climate change. But high production can also be classed as *high* vulnerability where there is a reliance on a single sector or species (i.e. low diversity or evenness in the species produced), which can increase exposure to climatic factors and vulnerability [20]. This has been illustrated in other studies where diversified food systems have been identified as having the potential to respond to high climate impacts, such as the fisheries-dependent countries Norway, Denmark, Ireland and the UK [27].

### (b) Regional trends

Effective regional policies that target key, often systemic, vulnerabilities and build on unique prospects will be needed to make progress on the interlinked challenges and inherent trade-offs that countries face in addressing climate change [56,57]. Regional classifications and overall rankings of aggregated vulnerability and leverage opportunity scores across all indicators identified Northern America and Europe as regions with comparably lower vulnerability and higher opportunity to leverage gains from mitigating approaches, these regions ranking 1st and 2nd respectively for both categories (figure 3*a,b* and table 2). These regions (and many of the countries therein; see electronic supplementary information, figure S1 and figure S2) had less vulnerable mariculture industries due to greater production quantities, less variance in production and higher diversity and evenness across the range of species cultured. Vulnerability in mariculture can also be associated with the effectiveness of governance at multiple spatial scales [15,28]. Northern America and Europe exhibited higher rankings for human development, and so while threats from climate change exist, their capacity to withstand and respond to these threats may be greater. This result is consistent with the known importance of socio-economic factors in aquaculture, especially governance and regulation, which influence whether countries engage with mariculture and its sustainable development [49,50]. Using existing platforms of good governance to build momentum for global, regional and local applications to enhance industry or sector-wide transformative capacity could be a way to support industry and communities to move beyond solely coping strategies in response to climate change and engage more easily with adaptive and transformative approaches, which will increase resilience [30].

**Table 2.** Median regional and global, vulnerability and leverage indicator scores (minimum and maximum values provided in parentheses).

(1) vulnerability indicators					
	1.1.1 mean annual production (*t*)	1.1.2 variance in annual production (% of mean)	1.1.3 mean annual production (*t* *per capita*)	1.1.4 diversity of species produced (Shannon's *H*)	1.1.5 evenness of species produced (Pielou's *J*)
Africa	372.3 (0–130.4 K)	46.8 (0–214)	<0.0001 (0–0.001)	4 (1–9)	0.2 (0–0.6)
Asia	167475 (21–29.8 M)	15.5 (0–77)	0.002 (<0.0001–0.04)	10.5 (1–44)	0.3 (0–0.7)
Europe	8792 (0–1.26 M)	18.5 (4.9–108)	0.0013 (0–1.6)	6 (1–50)	0.2 (0–0.5)
Latin America and the Caribbean	113.9 (0–1 M)	30 (0–283)	0.0001 (0–0.06)	2 (1–18)	0 (0–0.5)
Near East	50 (0–20.6 K)	90.5 (45.8–283)	<0.0001 (0–0.001)	2 (1–9)	0.2 (0–0.6)
Northern America	166730.9 (20–184 K)	11.3 (6.8–166)	0.0034 (0.001–0.01)	10 (2–20)	0.4 (0.1–0.5)
Southwest Pacific	92.1 (0–105 K)	35.2 (0–283)	0.0003 (0–0.04)	3 (1–11)	0.02 (0–0.7)
*Global*	*1506 (0–29.8 M)*	*26.6* (*0–283)*	*<0.0001* (*0–1.6)*	*4* (*1–50)*	*0.2* (*0–0.7)*
	1.2.1 mean annual seafood consumption (kg) *per capita*	1.2.2 variance in annual seafood consumption (kg) *per capita* (% of mean)	1.3.1 projected climate change impact–bivalves (proportion decline)	1.3.2 projected climate change impact–finfish (proportion decline)	1.4.1 Human Development Index score 2017
Africa	11.9 (0.2–29.8)	6.1 (1.4–24.2)	0.4 (0–1.8)	0.5 (0–1.4)	0.5 (0.4–0.8)
Asia	23.7 (0.5–188.6)	3.8 (0.5–16.3)	1 (0–1.8)	1.1 (0.4–2)	0.7 (0.6–0.9)
Europe	16.7 (3.6–87.9)	3.4 (0.3–18.4)	1 (0.4–2)	1.2 (0–2)	0.9 (0.8–1)
Latin America and the Caribbean	11.2 (0.9–53.5)	5 (1.2–200)	0.3 (0–1.8)	0.6 (0–1.5)	0.8 (0.5–0.8)
Near East	6.9 (0.2–25.3)	10.2 (2.2–34.6)	NA	0.8 (0–1.8)	0.7 (0.5–0.9)
Northern America	17.5 (16.8–18.2)	3.5 (2.6–4.4)	0.9 (0.9–1)	1.3 (0.7–1.5)	0.9 (0.9–0.9)
Southwest Pacific	34.7 (23.2–74)	2.1 (0.7–5.6)	0.6 (0.1–0.9)	0.8 (0.2–1.7)	0.7 (0.5–0.9)
*Global*	*15.7* (*0.2–188.6)*	*4.2* (*0.3–200)*	*0.8* (*0–2)*	*0.8* (*0–2)*	*0.8* (*0.4–1)*

	1.5.1 impact from coastal eutrophication (trend index)	1.5.2 GFSI, Natural Resources and Resilience score			
Africa	0.0002 (0–0.01)	57 (45–68)			
Asia	0.002 (0–0.02)	56 (44–72)			
Europe	0.008 (0.0001–16)	73 (50–82)			
Latin America and the Caribbean	0.0004 (0–0.01)	58 (43–75)			
Near East	0.0003 (0–1)	51 (41–64)			
Northern America	0.001 (0.0004–0.001)	69.5 (65–74)			
Southwest Pacific	0 (0–0.0001)	67.5 (63–72)			
*Global*	*0.0002* (*0–16)*	*59* (*41–82)*			
(2) leverage indicators					
	2.1.1 opportunity to reduce CO_2_ emissions (*t* per year *per capita*)	2.1.2 opportunity to reduce seafood consumption footprint (kg *per capita*)	2.1.3 opportunity to reduce proportion of imported seafood (ratio)	2.2.1 potential for restorative aquaculture–seaweed (index)	2.2.2 potential for restorative aquaculture–bivalves (index)
Africa	0.6 (0.05–8.7)	4.6 (1–36)	0.0002 (−2102.7–3.6)	34.9 (21.8–58.9)	32.8 (20.7–59.4)
Asia	2 (0.34–22.7)	25.7 (1–78.5)	−0.0263 (−279.2–334)	50.8 (33.7–63.7)	53.9 (46.4–63)
Europe	6.3 (0.53–14)	14.8 (1–66.6)	0.0098 (−18.6–14.3)	55.4 (33.5–73.7)	59.4 (33–71.4)
Latin America and the Caribbean	2.5 (0.25–33.3)	1 (1–28.9)	−0.0014 (−122.1–76)	36.6 (29.4–63.7)	46.2 (30.9–65.5)
Near East	4.6 (0.05–44)	1 (1–28.8)	−0.0012 (−0.2–3.1)	40.8 (17.5–49.3)	36.2 (15.6–51.8)
Northern America	16.6 (15.94–17.3)	23.6 (22.9–24.2)	0.2992 (0–0.6)	58.2 (58.2–58.2)	64 (62.5–65.5)
Southwest Pacific	1.1 (0–17.4)	1 (1–27.5)	0.1565 (−108.7–111.8)	20.6 (16.7–61.1)	24.4 (11.8–69.7)
*Global*	*2.7* (*0–44)*	*1* (*1–78.5)*	*0.0028* (−*2102.7–334)*	*43.8* (*16.7–73.7)*	*46.4* (*11.8–71.4)*
	2.3.1 opportunity to increase production from low GHG sectors (% production from low GHG sectors)	2.4.1 Regulatory Quality (index)	2.4.2 Logistics Performance (index)	2.4.3 research & development investment (% GDP)	
Africa	64.3 (0–100)	23.6 (1.9–81.7)	2.5 (2.1–3.4)	0.38 (0.05–0.79)	
Asia	33.2 (0–100)	48.1 (0–100)	3.2 (2.3–4)	0.49 (0.03–4.08)	
Europe	20 (0–100)	82 (31.7–99)	3.3 (2.4–4.2)	1.26 (0.24–4.21)	
Latin America and the Caribbean	0.3 (0–100)	57.9 (0.5–89.4)	2.7 (2.1–3.6)	0.25 (0.01–1.21)	
Near East	0 (0–0.1)	24.5 (1–79.8)	2.7 (2.1–4)	0.28 (0.01–0.87)	
Northern America	55.9 (23.7–88.2)	93 (92.3–93.8)	3.8 (3.7–3.9)	2.23 (1.72–2.75)	
Southwest Pacific	20.4 (0–100)	35.8 (10.1–98.6)	2.7 (2.2–3.9)	1.21 (0.03–2.18)	
*Global*	*12.6* (*0–100)*	*51* (*0–100)*	*2.8* (*2.1–4.2)*	*0.56* (*0.01–4.21)*	

Focusing on the role of more economically developed regions and countries in climate mitigation is warranted. Low-income countries tend to emit less GHGs than their share by population, while upper-middle and high-income countries emit more, some significantly more, such as the USA and Australia [43]. Yet, in pursuing climate-smart mariculture, regions and countries with apparently higher vulnerability and lower opportunity for leverage (as assessed here) must not be overlooked; those most at risk to the impacts of climate change are often those with the least capacity to respond [58]. Our analysis highlights challenges for the Near East and Africa especially, the Near East ranking as most vulnerable to climate change and 5th for leverage, and Africa as 6th for leverage and 5th for vulnerability, alongside Latin America and the Caribbean (figure 3*a*). These rankings largely reflect the less developed and more variable status of mariculture in these areas and high vulnerability according to the GFSI and HDI. Across all leverage indicators, these regions had low median leverage opportunity (figure 3*b*), despite individual countries within each region having opportunities associated with a range of specific pathways (see electronic supplementary information, figure S1). In particular, despite having substantial suitable area for mariculture in Africa [21] mariculture in this region faces increasing constraints as a result of competition with agriculture and a lack of availability of resources to support both industries [59]. This pressure will likely be exacerbated by the effects of climate change [27]. Also, countries in the Near East region such as Qatar, Kuwait, Bahrain and the United Arab Emirates have some of the world's highest rates of GHG emissions *per capita*, but these countries also have some of the least developed mariculture industry and currently nominal production in low GHG emissions sectors (i.e. bivalves and seaweed). Investment into technological or nature-based solutions to climate change via mariculture will likely first need to address key development issues, such as R&D into species best suited to local environmental conditions (additional description of regional-scale trends and potential policy implications is provided in the electronic supplementary results and country-scale scores grouped by regions are displayed for vulnerability in the electronic supplementary information, figure S1 and leverage, figure S2).

### (c) Implementing climate-smart strategies

Food industries that seek to be more resilient to climate change while synonymously pursuing economic outcomes can be viewed as ‘climate-smart’ [60]. Climate-smart aquaculture is an approach that ‘addresses the triple challenges of increasing productivity and adapting to climate change while reducing or removing greenhouse gas emissions (mitigation), where possible’ [61]. The strategies assessed here are already under discussion or in development in some geographies [53]. But their broader-scale adoption will be affected by the policies and capacity of national governments and their collaboration with industry and community. Political will is needed to engage with the transformative change that is required to halt the likely extreme impacts of climate change [58]. In climate-smart mariculture this could be influenced by the development of appropriate incentives, such as food or climate taxes and subsidies or fostering market-based motivations [6,53]. Incentives could be directly linked to GHG emissions reduction targets (e.g. our indicator 2.1.1) or to broader sustainability actions that might enhance or reinstate lost natural capital. Examples of the latter include the potential for mariculture to restore water filtration and nutrient extraction via bivalve or seaweed culture (2.2.1 and 2.2.2), which can support the preservation or restoration of marine habitats that sequester and store carbon, especially seagrasses, mangroves and salt marshes [26]. Market factors will also play a role in the efficacy of climate-smart strategies linked to dietary preferences. For example, as a comparatively low-cost and low-GHG emissions source of protein, bivalves could be a significant contributor to food security [62]. But shifting food production from higher-GHG emissions sources (2.3.1) to these products will require changes in consumption and demand, which might require the influence of a ‘price mechanism’ (i.e. an economic stimulus [6]) or government investment into the development of local markets. Market-based developments could be used to drive change through multiple pathways, an example here being supporting a reduction in long seafood supply chains (2.1.2 and 2.1.3).

Climate-smart pathways could also have pros and cons associated with the environmental setting in which they are applied, and they could still generate environmental impacts despite having a benefit for climate change mitigation. Implementation of mitigation strategies linked to mariculture must therefore include an understanding of habitat suitability (for sectors or species) under projected climate change (1.3.1, 1.3.2 and 1.5.2, also [21,22,63]) and work to address persistent negative impacts from industry activity, such as the occurrence of pathogens and pests, the introduction of non-native species to new areas, and impacts to water quality [64]. The contribution that mariculture could make to cumulative environmental stress, especially coastal pollution and eutrophication, should also be viewed ‘in light of’ the impacts these factors have on the industry itself [24] (1.5.1). Localized impacts *to* mariculture will also need to be considered, particularly the potential for higher mortalities and antimicrobial resistance in tropical areas [65]. In some areas mariculture might conceptually be able to return a benefit in climate mitigation, e.g. through the production of a high volume of seaweeds, but the negative effects of increasing production in certain species could risk exacerbating the prevalence of diseases or pathogens. Adopting an ecosystem approach to mariculture [66] and pursuing aquatic biosecurity using a ‘One Health’ lens, which aligns aquatic health to a broader set of metrics including animal welfare and human health [67], will be critical in responding to the manifold effects of climate change.

In implementing approaches to climate change mitigation, it must also be recognized that there is a risk of displacing climate-related impacts across sectors or locations. For example, production of popular finfish species such as salmonids can be more energy intensive in land- than sea-based systems [68]. As such, the effects of expanding production on land, in an attempt to lessen nutrient-based impacts on marine habitats and species, could result in increasing GHG emissions [69]. The nature of such trade-offs will be influenced by local factors, including the availability of renewable energy and biofuels, the type and quality of infrastructure and the source of supporting services and their energy intensity. The effects of local factors are illustrated by the potential to increase production from lower GHG emissions sectors (2.3.1). While these sectors typically generate lower levels of GHG emissions than others [4], excessive transport (e.g. to and from a hatchery, processing or market facilities located large distances from production sites) and energy-intensive facilities could undermine this value unless renewable energy sources are used [69]. Displacement of climate-related impacts could also occur in the expansion or transition of mariculture feed. Decoupling fed finfish mariculture from wild fisheries would increase fisheries sustainability, but shift pressure onto agriculture until gaps in technology for production of alternatives can be addressed [6,53,70]. Any increase in demand for land-based feed is likely to increase GHG emissions from land-use change, the use of water resources and infrastructure, and operations [1,2]. Furthermore, predicted increases in the frequency or severity of drought and flooding are highly likely to negatively impact land-based food production. These impacts will create additional, interlinked challenges for mariculture that is reliant on land-based crops [27,71].

Unintended consequences could also be generated by rushed or inappropriate development of mariculture to increase food and nutritional security where alternatives may be more immediately available. In particular, improved management of fisheries to address overexploitation and the effects of climate change could enhance fishing yields and profits under a range of predicted climate scenarios [6,72], and in some cases may be more appropriate than scaling-up mariculture production. Also, as they do in fisheries, traditional owners and small-scale operators in aquaculture play a vital role in food security, and there is a pressing need to increase the recognition and visibility of these actors in aquatic food systems [73]. Data currently available for aquaculture largely reflect an industrialized view of this food production system, and because of the reliance on this data in our assessment our analysis perpetuates this (industrialized) perspective. An important step in the development of climate-smart mariculture will be the inclusion of statistics at a sub-national scale, including locally contextualized data such as rates of participation in and production from mariculture by traditional owners. Ensuring that the participation of traditional owners in current but also past production is appropriately reflected in global datasets will support more effective and equitable outcomes. Here, addressing the perennial gap of low or inconsistent reporting across seafood industries and sustained misrepresentation of mariculture statistics [14,33,36], and broadening this base of knowledge to be more inclusive of the contribution of small-scale operators in particular, will increase accessibility to data at successively finer scales and enable all actors to share knowledge and discoveries for effective development of climate-smart approaches. Regions particularly affected by data deficiencies in our assessment were the Southwest Pacific, Africa and the Near East (see electronic supplementary information, figures S1 and S2), which, as we highlight above, are home to countries that will be most affected by climate change and potentially have the lowest capacity to respond.

## 4. Conclusion

Global attention and investment from those best placed to deliver substantial gains from climate mitigation solutions could yield high-impact results: results that will have flow-on benefits for countries most at risk from the impacts of climate change. While we found that opportunities exist to leverage climate outcomes associated with mariculture throughout most of the world—and that these opportunities must be fostered to ensure the drivers of climate change can be alleviated and the resilience of industry and communities increased—Northern America, Europe and a small cohort of countries within and outside of these regions, such as Australia, do have a considerable impact on global GHG emissions. These same regions and countries have comparatively higher opportunity for leverage combined with comparatively lower vulnerability. Consequently, their investment into the immediate development and adoption of climate-smart mariculture strategies could be influential, in-so-far as delivering the greatest gains in climate mitigation, but also in motivating political and industry will for broader and effective change.

## Data Availability

Electronic supplementary material, information is associated with this study and can be found in the online version. All data and materials associated with this study are available via FigShare at https://figshare.com/s/56a710deff3325c15575.

## References

[1] Hilborn R, Banobi J, Hall SJ, Pucylowski T, Walsworth TE. 2018 The environmental cost of animal source foods. Front. Ecol. Environ. **16**, 329-335. (10.1002/fee.1822)

[2] Poore J, Nemecek T. 2018 Reducing food's environmental impacts through producers and consumers. Science **360**, 987-992. (10.1126/science.aaq0216) 29853680

[3] Whitfield S, Challinor AJ, Rees RM. 2018 Frontiers in climate smart food systems: outlining the research space. Front. Sustain. Food Syst. **2**, 2. (10.3389/fsufs.2018.00002)

[4] Hall SJ, Delaporte MJ, Phillips MJ, Beveridge M, O'Keefe M. 2011 Blue frontiers: managing the environmental costs of aquaculture. Penang, Malaysia: The World Fish Centre.

[5] MacLeod MJ, Hasan MR, Robb DHF, Mamun-Ur-Rashid M. 2020 Quantifying greenhouse gas emissions from global aquaculture. Sci. Rep. **10**, 11679. (10.1038/s41598-020-68231-8) 32669630 PMC7363927

[6] Costello C et al. 2020 The future of food from the sea. Nature **588**, 95-100. (10.1038/s41586-020-2616-y) 32814903

[7] Pradeepkiran JA. 2019 Aquaculture role in global food security with nutritional value: a review. Transl. Anim. Sci. **3**, 903-910. (10.1093/tas/txz012) 32704855 PMC7200472

[8] Springmann M et al. 2018 Options for keeping the food system within environmental limits. Nature **562**, 519-525. (10.1038/s41586-018-0594-0) 30305731

[9] Duarte CM, Wu J, Xiao X, Bruhn A, Krause-Jensen D. 2017 Can seaweed farming play a role in climate change mitigation and adaptation? Front. Mar. Sci. **4**, 100. (10.3389/fmars.2017.00100)

[10] Laurens LML, Lane M, Nelson RS. 2020 Sustainable seaweed biotechnology solutions for carbon capture, composition, and deconstruction. Trends Biotechnol. **38**, 1232-1244. (10.1016/j.tibtech.2020.03.015) 32386971

[11] Cottrell RS et al. 2019 Food production shocks across land and sea. Nat. Sustain. **2**, 130-137. (10.1038/s41893-018-0210-1)

[12] Gaines S, Cabral R, Free CM. 2019 The expected impacts of climate change on the ocean economy. Washington, DC: World Resources Institute.

[13] Weatherdon LV, Magnan AK, Rogers AD, Sumaila UR, Cheung WWL. 2016 Observed and projected impacts of climate change on marine fisheries, aquaculture, coastal tourism, and human health: an update. Front. Mar. Sci. **3**, 48. (10.3389/fmars.2016.00048)

[14] Handisyde N, Telfer TC, Ross LG. 2017 Vulnerability of aquaculture-related livelihoods to changing climate at the global scale. Fish Fish. **18**, 466-488. (10.1111/faf.12186)

[15] Stewart-Sinclair PJ, Last KS, Payne BL, Wilding TA. 2020 A global assessment of the vulnerability of shellfish aquaculture to climate change and ocean acidification. Ecol. Evol. **10**, 3518-3534. (10.1002/ece3.6149) 32274006 PMC7141013

[16] Soto D, León-Muñoz J, Dresdner J, Luengo C, Tapia FJ, Garreaud R. 2019 Salmon farming vulnerability to climate change in southern Chile: understanding the biophysical, socioeconomic and governance links. Rev. Aquacult. **11**, 354-374. (10.1111/raq.12336)

[17] Maulu S, Hasimuna OJ, Haambiya LH, Monde C, Musuka CG, Makorwa TH, Munganga BP, Phiri KJ, Nsekanabo JD. 2021 Climate change effects on aquaculture production: sustainability implications, mitigation, and adaptations. Front. Sustain. Food Syst. **5**, 70. (10.3389/fsufs.2021.609097)

[18] Barange M, Bahri T, Beveridge MCM, Cochrane KL, Funge-Smith S, Poulain F. 2018 Impacts of climate change on fisheries and aquaculture. Synthesis of current knowledge, adaptation and mitigation options. FAO fisheries and aquaculture technical paper 627. Rome, Italy: Food and Agriculture Organization.

[19] Gephart JA, Rovenskaya E, Dieckmann U, Pace M, Brännström AA. 2016 Vulnerability to shocks in the global seafood trade network. Environ. Res. Lett. **11**, 035008. (10.1088/1748-9326/11/3/035008)

[20] Troell M et al*.* 2014 Does aquaculture add resilience to the global food system? Proc. Natl Acad. Sci. USA **111**, 13257. (10.1073/pnas.1404067111) 25136111 PMC4169979

[21] Oyinlola MA, Reygondeau G, Wabnitz CCC, Troell M, Cheung WWL. 2018 Global estimation of areas with suitable environmental conditions for mariculture species. PLoS ONE **13**, e0191086. (10.1371/journal.pone.0191086) 29357374 PMC5774971

[22] Gentry RR, Froehlich HE, Grimm D, Kareiva P, Parke M, Rust M, Gaines SD, Halpern BS. 2017 Mapping the global potential for marine aquaculture. Nat. Ecol. Evol. **1**, 1317-1324. (10.1038/s41559-017-0257-9) 29046547

[23] Gephart JA, Pace ML. 2015 Structure and evolution of the global seafood trade network. Environ. Res. Lett. **10**, 125014. (10.1088/1748-9326/10/12/125014)

[24] Halpern BS et al*.* 2019 Spatial and temporal changes in cumulative human impacts on the world's ocean. Nat. Commun. **6**, 7615. (10.1038/ncomms8615) PMC4510691 26172980

[25] Theuerkauf SJ, Morris JA, Waters TJ, Wickliffe LC, Alleway HK, Jones RC. 2019 A global spatial analysis reveals where marine aquaculture can benefit nature and people. PLoS ONE **14**, e0222282. (10.1371/journal.pone.0222282) 31596860 PMC6784979

[26] Petersen JK, Saurel C, Nielsen P, Timmermann K. 2016 The use of shellfish for eutrophication control. Aquacult. Int. **24**, 857-878. (10.1007/s10499-015-9953-0)

[27] Blanchard JL et al. 2017 Linked sustainability challenges and trade-offs among fisheries, aquaculture and agriculture. Nat. Ecol. Evol. **1**, 1240-1249. (10.1038/s41559-017-0258-8) 29046559

[28] Soto D, Ross LG, Handisyde N, Bueno PB, Beveridge MCM, Dabbadie L, Aguilar-Manjarrez J, Cai J, Pongthanapanich T. 2018 Chapter 21: Climate change and aquaculture: vulnerability and adaptation options. In Impacts of climate change on fisheries and aquaculture. Synthesis of current knowledge, adaptation and mitigation options (eds M Barange, T Bahri, MCM Beveridge, KL Cochrane, S Funge-Smith), pp. 465-490. Rome, Italy: Food and Agriculture Organization.

[29] Béné C. 2020 Resilience of local food systems and links to food security – a review of some important concepts in the context of COVID-19 and other shocks. Food Secur. **12**, 805-822. (10.1007/s12571-020-01076-1) 32837646 PMC7351643

[30] Béné C, Headey D, Haddad L, von Grebmer K. 2016 Is resilience a useful concept in the context of food security and nutrition programmes? Some conceptual and practical considerations. Food Secur. **8**, 123-138. (10.1007/s12571-015-0526-x)

[31] Dorninger C et al. 2020 Leverage points for sustainability transformation: a review on interventions in food and energy systems. Ecol. Econ. **171**, 106570. (10.1016/j.ecolecon.2019.106570)

[32] West PC et al. 2014 Leverage points for improving global food security and the environment. Science **345**, 325-328. (10.1126/science.1246067) 25035492

[33] Halpern BS et al. 2019 Opinion. Putting all foods on the same table: achieving sustainable food systems requires full accounting. Proc. Natl Acad. Sci. USA **116**, 18 152-18 156. (10.1073/pnas.1913308116) PMC6744877 31506376

[34] Tlutsy MF et al. 2019 Reframing the sustainable seafood narrative. Glob. Environ. Change **59**, 101991. (10.1016/j.gloenvcha.2019.101991)

[35] Clark MA, Springmann M, Hill J, Tilman D. 2019 Multiple health and environmental impacts of foods. Proc. Natl Acad. Sci. USA **116**, 23 357-23 362. (10.1073/pnas.1906908116) 31659030 PMC6859310

[36] Edwards P, Zhang W, Belton B, Little DC. 2019 Misunderstandings, myths and mantras in aquaculture: its contribution to world food supplies has been systematically over reported. Mar. Policy **106**, 103547. (10.1016/j.marpol.2019.103547)

[37] FAO. 2020 FishStatJ - software for fishery and aquaculture statistical time series. Rome. See http://www.fao.org/fishery/statistics/software/fishstatj/en.

[38] Froehlich HE, Gentry RR, Halpern BS. 2018 Global change in marine aquaculture production potential under climate change. Nat. Ecol. Evol. **2**, 1745-1750. (10.1038/s41559-018-0669-1) 30201967

[39] FAO. 2020. FAOSTAT, ‘New Food Balances’. See http://www.fao.org/faostat/en/#data/FBS.

[40] United Nations. 2020 Human Development Index (HDI). See http://hdr.undp.org/en/content/human-development-index-hdi.

[41] Hoekstra J, Molnar JL, Jennings M, Revenga C, Spalding MD, Boucher TM, Robertson JC, Heibel TJ, Ellison K. 2010 The atlas of global conservation: changes, challenges, and opportunities to make a difference. Oakland, CA: University of California Press.

[42] The Economist Group. 2020 Global Food Security Index. See https://impact.economist.com/sustainability/project/food-security-index/.

[43] The World Bank. 2020 DataBank. See https://data.worldbank.org/.

[44] Guillen J et al*.* 2019 Global seafood consumption footprint. Ambio **48**, 111-122. (10.1007/s13280-018-1060-9) 29845576 PMC6346599

[45] The World Bank. 2018 Regulatory quality. World Governance Indicators. See https://info.worldbank.org/governance/wgi/.

[46] Shannon CEA. 1948 A mathematical theory of communication. Bell Syst. Tech. J. **27**, 379-423. (10.1002/j.1538-7305.1948.tb01338.x)

[47] Pielou EC. 1966 Species-diversity and pattern-diversity in the study of ecological succession. J. Theor. Biol. **10**, 370-383. (10.1016/0022-5193(66)90133-0) 5964400

[48] Clune S, Crossin E, Verghese K. 2017 Systematic review of greenhouse gas emissions for different fresh food categories. J. Clean. Prod. **140**, 766-783. (10.1016/j.jclepro.2016.04.082)

[49] Gentry RR, Ruff EO, Lester SE. 2019 Temporal patterns of adoption of mariculture innovation globally. Nat. Sustain. **2**, 949-956. (10.1038/s41893-019-0395-y)

[50] Ruff EO, Gentry RR, Lester SE. 2020 Understanding the role of socioeconomic and governance conditions in country-level marine aquaculture production. Environ. Res. Lett. **15**, 1040a8. (10.1088/1748-9326/abb908)

[51] R Core Team. 2019 R: a language and environment for statistical computing. Vienna, Austria: R Foundation for Statistical Computing.

[52] Béné C, Oosterveer P, Lamotte L, Brouwer ID, de Haan S, Prager SD, Talsma EF, Khoury CK. 2019 When food systems meet sustainability – current narratives and implications for actions. World Dev. **113**, 116-130. (10.1016/j.worlddev.2018.08.011)

[53] Costello C, Cao L. 2019 The future of food from the Sea. Washington, DC: World Resources Institute.

[54] Nyström M, Jouffray J-B, Norström AV, Crona B, Søgaard Jørgensen P, Carpenter SR, Bodin Ö, Galaz V, Folke C. 2019 Anatomy and resilience of the global production ecosystem. Nature **575**, 98-108. (10.1038/s41586-019-1712-3) 31695208

[55] Ritchie H, Roser M, Rosado P. 2020 CO_2_ and greenhouse gas emissions. Published online at OurWorldInData.org. Retrieved from: https://ourworldindata.org/co2‐and‐other‐greenhouse‐gasemissions.

[56] Ingram J. 2011 A food systems approach to researching food security and its interactions with global environmental change. Food Sec. **3**, 417-431. (10.1007/s12571-011-0149-9)

[57] Nilsson M, Griggs D, Visbeck M. 2016 Policy: map the interactions between sustainable development goals. Nature **534**, 320-322. (10.1038/534320a) 27306173

[58] IPCC. 2019 *IPCC special report of the ocean and cryosphere in a changing climate* (eds H‐O Pörtner *et al*.). See https://www.ipcc.ch/srocc/.

[59] Nyamete F, Chacha M, Msagati T, Raymond J. 2020 Prospects for aquaculture development in Africa in the context of a changing climate: a review. Int. J. Biosci. **17**, 1-13. (10.12692/ijb/17.4.1-31)

[60] Saj S, Torquebiau E, Hainzelin E, Pages J, Maraux F. 2017 The way forward: an agroecological perspective for Climate-Smart Agriculture. Agr. Ecosyst. Environ. **250**, 20-24. (10.1016/j.agee.2017.09.003)

[61] FAO. 2018 The state of world fisheries and aquaculture. Meeting the Sustainable Development Goals. Rome, Italy: Food and Agriculture Organization.

[62] Ray NE, Maguire TJ, Al-Haj AN, Henning MC, Fulweiler RW. 2019 Low greenhouse gas emissions from oyster aquaculture. Environ. Sci. Technol. **53**, 9118-9127. (10.1021/acs.est.9b02965) 31295406

[63] Klinger DH, Levin SA, Watson JR. 2017 The growth of finfish in global open-ocean aquaculture under climate change. Proc. R. Soc. B **284**, 20170834. (10.1098/rspb.2017.0834) PMC5647286 28978724

[64] Naylor RL et al*.* 2021 A 20-year retrospective review of global aquaculture. Nature **591**, 551-563. (10.1038/s41586-021-03308-6) 33762770

[65] Reverter M et al*.* 2020 Aquaculture at the crossroads of global warming and antimicrobial resistance. Nat. Commun. **11**, 1870. (10.1038/s41467-020-15735-6) 32312964 PMC7170852

[66] Brugère C, Aguilar-Manjarrez J, Beveridge MCM, Soto D. 2019 The ecosystem approach to aquaculture 10 years on – a critical review and consideration of its future role in blue growth. Rev. Aquacult. **11**, 493-514. (10.1111/raq.12242)

[67] Stentiford GD et al*.* 2020 Sustainable aquaculture through the One Health lens. Nat. Food **1**, 468-474. (10.1038/s43016-020-0127-5) 37128071

[68] Bohnes FA, Hauschild MZ, Schlundt J, Laurent A. 2019 Life cycle assessments of aquaculture systems: a critical review of reported findings with recommendations for policy and system development. Rev. Aquacult. **11**, 1061-1079. (10.1111/raq.12280)

[69] Jones AR, Alleway HK, McAfee D, Reis-Santos P, Theuerkauf SJ, Jones RC. 2021 Climate-friendly seafood: the potential for emissions reduction and carbon capture in marine aquaculture. BioScience **72**, 123-143. (10.1093/biosci/biab126) PMC8824708 35145350

[70] Froehlich HE, Jacobsen NS, Essington TE, Clavelle T, Halpern BS. 2018 Avoiding the ecological limits of forage fish for fed aquaculture. Nat. Sustain. **1**, 298-303. (10.1038/s41893-018-0077-1)

[71] Ahmed N, Thompson S, Glaser M. 2019 Global aquaculture productivity, environmental sustainability, and climate change adaptability. Environ. Manage. **63**, 159-172. (10.1007/s00267-018-1117-3) 30460481

[72] Gaines SD et al*.* 2018 Improved fisheries management could offset many negative effects of climate change. Sci. Adv. **4**, eaao1378. (10.1126/sciadv.aao1378) 30167455 PMC6114984

[73] Short RE et al*.* 2021 Harnessing the diversity of small-scale actors is key to the future of aquatic food systems. Nat. Food **2**, 733-741. (10.1038/s43016-021-00363-0) 37117475

